# Amyloid proteotoxicity initiates an inflammatory response blocked by cannabinoids

**DOI:** 10.1038/npjamd.2016.12

**Published:** 2016-06-23

**Authors:** Antonio Currais, Oswald Quehenberger, Aaron M Armando, Daniel Daugherty, Pam Maher, David Schubert

**Affiliations:** 1 Cellular Neurobiology Laboratory, The Salk Institute for Biological Studies, La Jolla, CA, USA; 2 Department of Pharmacology, University of California San Diego, La Jolla, CA, USA; 3 Department of Medicine, University of California San Diego, La Jolla, CA, USA

## Abstract

The beta amyloid (Aβ) and other aggregating proteins in the brain increase with age and are frequently found within neurons. The mechanistic relationship between intracellular amyloid, aging and neurodegeneration is not, however, well understood. We use a proteotoxicity model based upon the inducible expression of Aβ in a human central nervous system nerve cell line to characterize a distinct form of nerve cell death caused by intracellular Aβ. It is shown that intracellular Aβ initiates a toxic inflammatory response leading to the cell's demise. Aβ induces the expression of multiple proinflammatory genes and an increase in both arachidonic acid and eicosanoids, including prostaglandins that are neuroprotective and leukotrienes that potentiate death. Cannabinoids such as tetrahydrocannabinol stimulate the removal of intraneuronal Aβ, block the inflammatory response, and are protective. Altogether these data show that there is a complex and likely autocatalytic inflammatory response within nerve cells caused by the accumulation of intracellular Aβ, and that this early form of proteotoxicity can be blocked by the activation of cannabinoid receptors.

## Introduction

Nerve cell death from the accumulation of aggregated or amyloid-like proteins is a common theme in most age-dependent neurodegenerative diseases. However, there are no drugs that significantly inhibit cell death associated with Alzheimer’s disease (AD), Parkinson’s or Huntington’s diseases. This could be because most interest has been in the late manifestations of the disease, not in the initial changes in cell metabolism that ultimately lead to nerve cell death.^1^ In the context of life span, slowing down the removal of aggregated proteins in the brains of flies shortens life span, while expediting their rate of removal extends life span.^2^ Therefore, it is likely that the accumulation of intracellular aggregated protein in the brain occurs throughout life, contributes to cognitive aging, and may also be involved in the initiation of many old age-associated diseases.

Although debated,^3,4^ the accumulation of intracellular amyloid beta (Aβ) is an early event in AD. In both humans and rodents, intracellular Aβ accumulation is observed well before extracellular amyloid.^5–8^ Similarly, both aggregated huntingtin and alpha synuclein are found in neurons before disease onset.^9,10^

As with the accumulation of intracellular proteins, central nervous system (CNS) inflammation is elevated with age and increases in disease.^11^As AD is associated with neuronal dysfunction, we hypothesized that proteotoxicity in nerve cells themselves may initiate an inflammatory response that can lead directly to their death and contribute to overall inflammation in the CNS. The following experiments identify the molecular basis of this inflammatory response using a human CNS nerve cell line that conditionally expresses Aβ.

## Results

**Figure 1:**
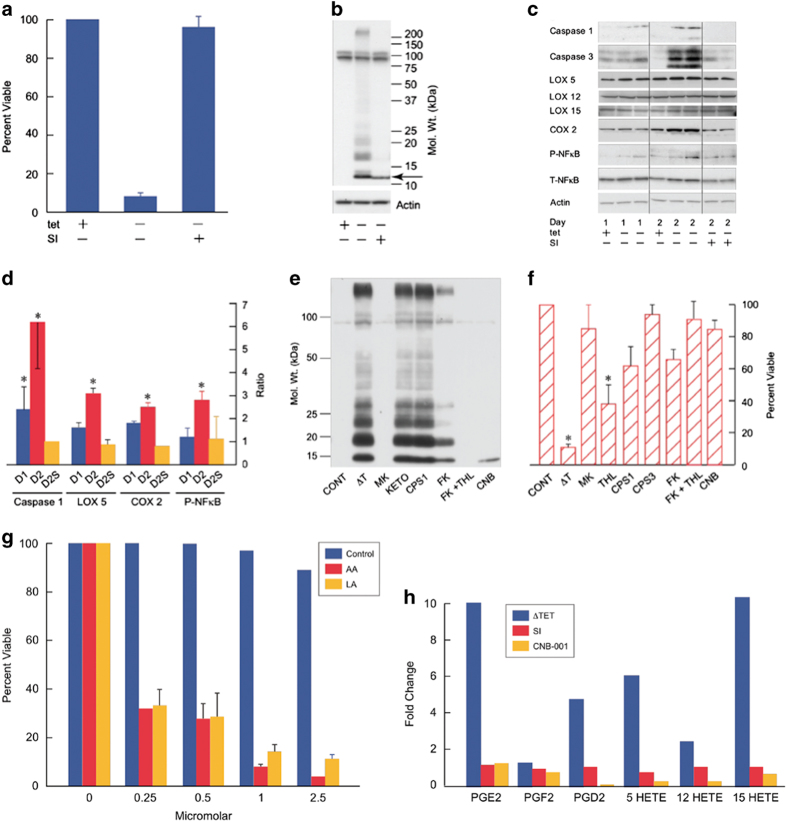
Intraneuronal Aβ induces an inflammatory response that is potentiated by arachidonic and linoleic acids. (**a**) MC65 cells were induced to make intracellular Aβ by the removal of tet (tet−) from the culture medium in the presence (SI+) or absence (SI−) of 10 μmol/l γ-secretase inhibitor 10 (Calbiochem) and cell death assayed on day 4. (**b**). Expression of intracellular Aβ using 6E10 antibody on day 2 in same conditions as in **a**. Arrow indicates C99 and the 100 kDa band is APP. (**c**) MC65 cells were induced to make Aβ (−tet) or uninduced (+tet) in the presence or absence of 10 μmol/l γ secretase inhibitor (SI) 1 and 2 days later proteins were assayed by western blotting. (**d**) Protein amount was quantified and normalized either to actin or in the case of phosphorylation to the total protein. D1=Day 1, D2=Day 2, D2S=Day 2+SI. (**e**) Western blot of Aβ two days after tet withdrawal (ΔT) in the presence of MK806 (MK, 1 μmol/l), ketorolac (KETO, 10 μmol/l), caspase 1 inhibitor (CPS 1, 50 μmol/l), FkG 11 (FK, 5 μmol/l), THL (5 μmol/l) or CNB-001 (CNB, 1 μmol/l). (**f**) Cells were incubated for 4 days in the presence of the caspase 1 inhibitor (CPS1) Z-YVAD-FMC (50 μmol/l), caspase 3 inhibitor (CPS3, 50 μmol/l), Z-DEVD-FMk (50 μmol/l) or the compounds in **e**. The percent viable cells is presented. *P<0.01, *n*=3–5. (**g**) MC65 cells were induced to make Aβ in the presence of increasing amounts of LA or AA and cell death was measured on day 2 instead of day 4 when the cells normally die. The control is cells without tet. All changes with AA or LA: *P*<0.01, *n*=4. (**h**) MC65 cells were grown in the absence of tet (ΔT) with 100 nmol/l J147 or 10 μmol/l γ secretase inhibitor (SI) and eicosanoid accumulation assayed in the culture medium 2 days later, and presented as fold change relative to uninduced cells. Baseline values (fmole/2×10^6^ cells) are: PGE2, 49; PGF2, 1315; PGD2, 257; 5-HETE, 11; 12 HETE, 101; 15 HETE, 28. These data are from a single experiment, but similar results were obtained in three independent experiments. The absolute amounts of extracellular eicosanoids varied, but the relative changes within the individual experiments were similar (see the separate experiment in Figure 2). APP, amyloid precursor protein; Aβ, beta amyloid.

MC65 cells are a human CNS nerve cell line that contains the C-99 fragment of the amyloid precursor protein under the control of a tetracycline (tet)-sensitive promoter.^12^ The parent cell line is SK-N-MC from a human brain tumor, and it has an electrically excitable membrane typical of neurons.^13^ When tet is withdrawn, cells express C-99 that is converted to Aβ by γ-secretase and the cells die within 4 days (Figure 1a,b). Aβ remains within the cell and forms aggregates.^12,14^ In the presence of γ-secretase inhibitors (SI), cells accumulate C-99, but do not die, and C99 does not aggregate.

### Intraneuronal Aβ induces the expression of proinflammatory molecules

Inflammation is associated with the elevated expression of cytokines and chemokines. To assay for the expression of these genes following the induction of Aβ in MC65 cells, the mRNA expression of 184 inflammation-associated genes was assayed sequentially for three days. Table 1 shows increases in the expression of 12 genes.

IL-8 expression is linked to late onset AD.^15^ Importantly, IL-8 crosses the blood brain barrier and stimulates the recruitment of immune cells into the brain.^16^ Aβ causes a 10-fold increase in IL-8 gene expression, and IL-8 is detected in the culture supernatant 2 days after the induction of Aβ (Table 1).

It was next asked whether the intracellular expression of Aβ leads to an increase in proinflammatory pathways. NFkB is a ubiquitous proinflammatory molecule whose activation includes phosphorylation. Phosphorylation of its p65 subunit is increased following the expression of Aβ in MC65 cells (Figure 1c,d). Inflammation is also associated with the activation of caspase 1. Figure 1c,d shows that following the expression of Aβ, caspase 1 is activated as defined by the appearance of activation-dependent cleavage products.

In cases of caspase-1 activation, cell death is ultimately caused by caspase-3.^17^ Figure 1c also shows that caspase-3 is strongly activated on day 2 following the start of Aβ accumulation. The caspase-1 inhibitor Z-YVAD-FMC (CPS 1) and the caspase-3 inhibitor Z-DEVD-FMK (CPS 3), had no effect on Aβ levels (Figure 1e and not shown), but reduced cell death (Figure 1f). All proinflammatory responses were blocked by a γ secretase inhibitor. Therefore, the expression of Aβ leads to the induction of an inflammatory pathway and caspase dependent cell death.

Eicosanoids are derivatives of arachidonic acid (AA) or related polyunsaturated fatty acids that have either pro- or anti-inflammatory effects. Leukotrienes are made by lipoxygenases (LOXs), while prostaglandins are synthesized by cyclooxygenases (COXs). COX 1 is constitutively expressed; COX 2 is inducible under conditions of stress.^18^ Figure 1c,d show that COX 2 expression is increased by 2 days.

In human cells, there are three LOXs.^5,12,15,19^ 5-LOX is abundantly expressed in neurons and is elevated in AD.^20^ Figure 1c,d show that 5-LOX increases following Aβ induction.

To determine whether LOX and COX activities are required for cell death, inhibitors were assayed for their ability to block toxicity. The COX inhibitors Ibuprofen and Indomethacin did not block toxicity, nor did the COX-2 inhibitor NS-398 or the generic COX inhibitor Ketorolac (Keto; Supplementary Table S1); none altered intracellular Aβ (Figure 1e). In contrast, the 5-LOX inhibitor CNB-001 (CNB)^14^ and the 5-LOX-activating protein inhibitor MK806 (MK) prevented the accumulation of Aβ and cell death (Figure 1e,f).

### Aβ-induced AA is derived from several lipases

Since the major substrate for LOXs and COXs is AA, it was asked whether AA potentiates cell death. When induced to make Aβ in the presence of AA, cells die in a dose-dependent manner in 2 days following Aβ expression rather than 4 days in control cultures (Figure 1g). Linoleic acid is a precursor to AA and also a substrate for LOXs, and it also potentiates toxicity (Figure 1g). These data suggest that AA may be produced following Aβ induction and that AA is a substrate for nerve cell death. Analysis of AA after 3 days of Aβ induction showed between a two- and threefold increase (2.5±0.6, *P*<0.001 *n*=5).

The physiology of a cell is very different when it is stressed (in this case by proteotoxicity) as compared with its normal state. Compounds that have no effect on non-stressed cells may engage pathways that are induced by stress and therefore their action may only be detected under stressful conditions. This is the case with extracellular AA and in the following experiments where the effect of pharmacological intervention is only observed in Aβ-induced cells.

Phospholipase A2 (PLA2) is the major source of AA in the brain and contributes to AD pathology.^21^ The three forms in human cells include Ca^++^ dependent (cPLA2), Ca^++^ independent (iPLA2) and secretory (sPLA2). *iPLA2* gene knockout AD mice have reduced amyloid plaque load and improved behavior.^22^ In humans, variants of the enzymes are risk factors for AD, and the enzymes that metabolize AA are increased in AD and AD mice.^21^ Most AA in the brain is derived from iPLA2, but there are other potential sources. MC65 cell death is partially prevented by the broad-spectrum phospholipase A2 inhibitors 4-octadecyl benzyl acrylic acid (OBAA) and chlorpromazine (Supplementary Table S1). FkGk11, an iPLA2-specific inhibitor, reduces toxicity and intracellular Aβ accumulation (Figure 1e,f), while the cPLA2 inhibitors methyl arachidonyl fluorophosphonate (MAFP), CAY10502, and pyrrophene have no effect (Supplementary Table S1). The sPLA2 inhibitor thioetheramide does not inhibit cell death at concentrations effective in human cells.^23^ Therefore, the PLA2 inhibitors are only partially effective in preventing cell death.

Alternative sources of AA are triacylglycerols. The monoacylglycerol lipase (MAGL) inhibitor JZL1 was inactive in reducing cell death, as was the inhibitor of diacylglycerol lipase, RHC-80287 (Supplementary Table S1). However, the generic lipase inhibitor CAY10499 and the triacylglycerol lipase inhibitors, tetrahydrolipstatin (THL) and Atglistin, partially blocked toxicity (Supplementary Table S1, Figure 1f). Although none of the lipase inhibitors individually completely blocked toxicity, the combination of FkGk11 and THL did (Figure 1f, Supplementary Table S1). In addition, this combination reduces the increase in AA following Aβ induction by (91±6%, *n*=3) and also blocks the accumulation of Aβ (Figure 1e). Because of the profound effect of AA, it was asked whether eicosanoids themselves have roles in the clearance of intracellular Aβ and nerve cell death.

### Intraneuronal Aβ increases eicosanoid production

**Figure 2:**
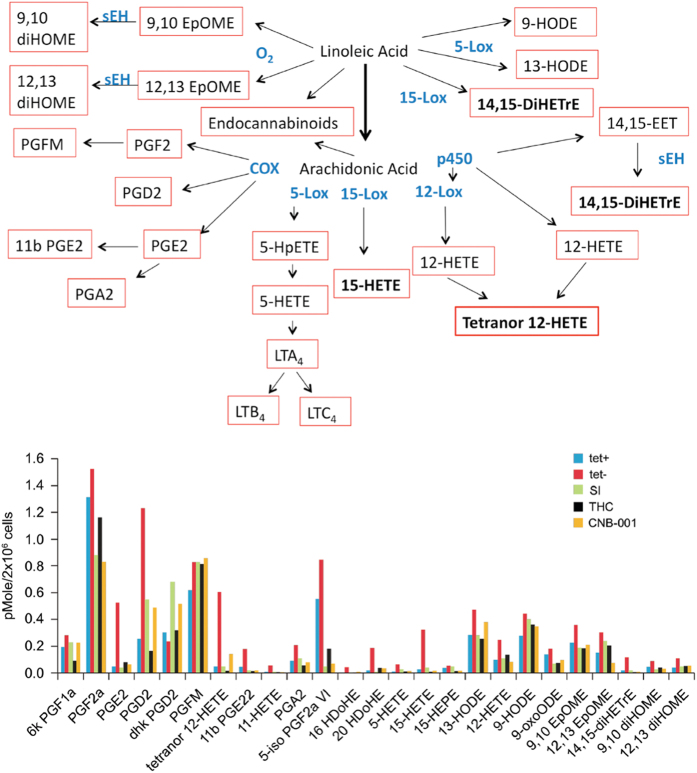
Schematic representation and GC/MS/MS analysis of the induction of eicosanoid synthesis following Aβ expression in MC65 cells. Cells were induced to make Aβ (−tet) or not (+tet) for 48 h and the culture supernatant assayed for eicosanoids. Of the 164 eicosanoids assayed, these were the only ones detected. In the same experiment, induced cells were treated with 1 μmol/l THC, 10 μmol/l Calbiochem secretase inhibitor Ten (SI), or 500 nmol/l CNB-001. These data are from a single experiment, and similar results were obtained in three independent experiments. Although the absolute amounts of individual eicosanoids in the culture supernatants were somewhat variable, the relative effects of SI, THC and CNB-001 were the same. Aβ, beta amyloid.

When cells are stressed, they initially mount a protective response. Twenty hrs after Aβ induction in MC65 cells, only increases in prostaglandins PGD_2_ and PGE_2_ can be found in the cell culture medium (not shown), but by 48 h there is a relatively simultaneous expression of multiple prostaglandins and leukotrienes that is blocked by a γ-secretase inhibitor or by CNB-001 (Figure 1h, Figure 2). The genesis of the eicosanoids that are elevated following Aβ induction is also shown.

### Prostaglandins and leukotrienes have opposing effects on cell death

The effect of the secreted eicosanoids on intracellular Aβ toxicity were examined in two ways. First, each eicosanoid identified in the culture medium was added to induced or uninduced cells using a twofold serial dilution between 20 μmol/l and 10 nmol/l, and it was determined whether the compound was either neuroprotective, potentiated toxicity or was directly toxic. Second, when possible, these data were confirmed by receptor antagonists or agonists.

**Figure 3:**
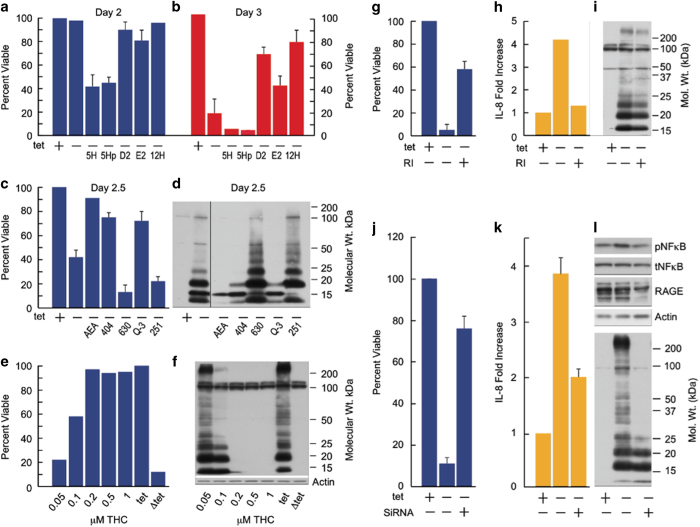
Eicosanoids, endocannabinoids and RAGE modulate Aβ toxicity. MC65 cells induced (Δtet) to make Aβ in the presence or absence of the indicated compounds and cell viability and/or intracellular Aβ levels monitored at day 2 or 2.5 following induction. 5H (5-HETE, 500 nmol/l); 5Hp (5-HETE peroxide, 500 nmol/l); 12H (12 HETE, 500 nmol/l); D2 (PGD2, 500 nmol/l); E2 (PDE2, 10 μmol/l); AEA (arachidonoyl ethanolamide, 1 μmol/l); THC (tetrahydrocannabinol, 50 nmol/l); Q-3 (Q-3 carboxamide, 100 nmol/l); 404 (AM404, 1 μmol/l); 630 (AM630, 10 nmol/l); 251 (AM251, 1 μmol/l). (**a**) Cell viability at 2 days; (**b**) cell viability at 3 days; (**c**) cell viability at 2.5 days; (**d**) Aβ at 2 days; (**e**) THC dose response, cell viability; (**f**) THC dose response, intracellular Aβ at 2 days. (**g**) MC65 cells were incubated with or without the RAGE inhibitor (RI) FPS-ZMII (1 μmol/l) with (+) and without (−) tet. Cell viability assayed on day 4 (**h**) The amount of IL8 assayed on day 3 following Aβ induction. (**i**) Aβ expression was monitored on day 3 (**j**) Cells were treated with RAGE siRNA or control siRNA and cell viability assayed after 2 days. (**k**) IL-8 was assayed on day 3, (**l**) Phospho NFkB, RAGE and Aβ expression were assayed on day 3. Aβ, beta amyloid; SiRNA, small interfering RNA.

Figure 3a,b and Supplementary Table S2 show that PGE2 and PGD2, are neuroprotective, whereas 5-HETE and its downstream metabolites LTA4 and LTB4 potentiate toxicity. Fifteen HETE is not reproducibly active, while 12 HETE is protective (Figure 3b). However, none of the prostaglandins alter intracellular Aβ levels at day 2, while 5-HETE stimulates Aβ accumulation (not shown). The other eicosanoids lack activity (Supplementary Table S2). Importantly, none of the eicosanoids were directly toxic to uninduced MC65 cells.

Prostaglandin and leukotrienes interact with a large number of receptors. PGE2 interacts with EP1–4. Supplementary Table S1 shows that the EP1 antagonist SC51089 and EP4 antagonists HA23848 and GW627368X all potentiate Aβ toxicity. The EP4 antagonists were 10-fold more potent, suggesting the primary involvement of EP4.

PGD2 signals through DP1 and DP2. It was asked whether the DP1-selective agonist BW245C or the selective DP2 agonist 15-PGJ(2) were neuroprotective. Only BW245C was effective (Supplementary Table S1), suggesting that PGD2 protection is via DP1.

There are a large number of molecular targets for the leukotrienes that are blocked by a γ-secretase inhibitor or by CNB-001. Of these, the LTB4 receptors BLT1 and BLT2 are the best studied. LTA4 and LTB4 potentiate Aβ toxicity themselves and the BLT antagonist LY255283 is weakly protective (Supplementary Tables S1 and S2).

### Cannabinoids remove intraneuronal Aβ

In addition to prostaglandins and leukotrienes, AA is a component of a very large family of endocannabinoids (ECs) that are, in turn, metabolized to AA. The EC arachidonoyl ethanol amide (AEA) is expressed in the brain.^24^ The major cannabinoid receptors are CB1 and CB2, and AEA activates both. The data in Supplementary Table S1 and Figure 3c,d show that AEA promotes MC65 survival and blocks intracellular Aβ accumulation, as do its hydrolysis-stable analogs arvanil (not shown) and AM404 (404) (Supplementary Table S1, Figure 3c,d). UBR597, an inhibitor of the enzyme that degrades AEA, is also protective (Supplementary Table S1) as is the CB2 agonist Q-3 (Figure 3c,d, Supplementary Table S1). Conversely, toxicity and Aβ accumulation are enhanced by CB1 and CB2 antagonists AM281, AM251 and AM630 (Supplementary Table S1, Figure 3c,d). A number of additional CB1 and CB2 agonists or antagonists were assayed, but no pharmacological distinction between CB1 and CB2 could be made (Supplementary Table S1). Of the compounds tested, THC is the most potent CB-1 agonist, with an EC_50_ below 50 nmol/l (Figure 3e,f). THC is protective, removes intraneuronal Aβ and completely eliminates the elevated eicosanoid production in induced MC65 cells.

### Inflammation is initiated in part via RAGE

Receptor for advanced glycation endproducts (RAGE) interacts with Aβ and can be expressed inside cells.^25^ As RAGE activation induces an inflammatory response, we asked whether RAGE is involved in Aβ-induced cell death. Figure 3g shows that a RAGE inhibitor, FPS-ZMI, partially blocks cell death, and Figure 3h shows that IL-8 secretion is also reduced by FPS-ZMI, as is intracellular Aβ (Figure 3i). Knocking down RAGE with siRNA further supported the results, showing a reduction in cell death, IL-8 secretion and Aβ accumulation (Figure 3j–l). The reduction of RAGE expression also blocked the activation of NFkB as determined by lowering its phosphorylation to baseline levels (Figure 3l), but it did not significantly reduce the expression of eicosanoids or alter AA levels (not shown).

### Extracellular proinflammatory cytokines also potentiate Aβ toxicity

**Figure 4:**
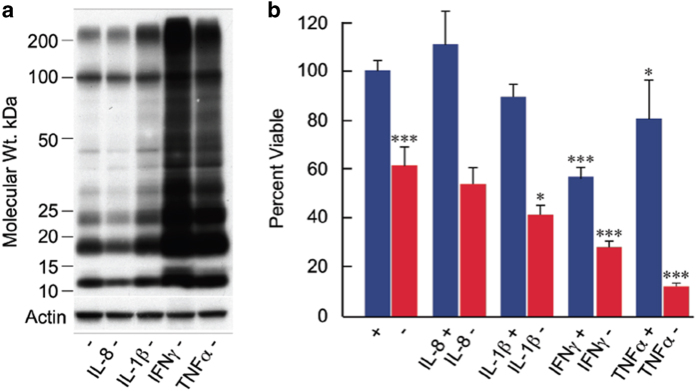
Cytokines potentiate toxicity and Aβ accumulation. MC65 cells were either uninduced (+) or induced (−) to make Aβ in the presence or absence of IL-8 (10 ng/ml), IL-1 (10 ng/ml), interferon γ (INF-γ; 10 ng/ml) or TNFα (10 ng/ml). Sixty hours later, intracellular Aβ was determined by western blotting (antibody 6E10) (**a**) and cell viability by the MTT assay (**b**) *n*=4; *P<0.05; **P<0.01, ***P<0.001. AA, arachidonic acid; Aβ, beta amyloid; IFN, interferon; IL, interleukin; TNF, tumor necrosis factor.

The above data show that the death of neurons may be progressively autocatalytic due to the production of a variety of proinflammatory molecules. It was therefore asked whether the cytokines enhance the rate of Aβ accumulation and death. MC65 cells were exposed to IL-8, IL-1β, IFNγ or TNFα for 2 days and intraneuronal Aβ and cell viability assayed. IFNγ and TNFα strongly potentiate both the accumulation of Aβ and cell death (Figure 4). IL-8 was ineffective, likely because large amounts are secreted and the cells are desensitized.

## Discussion

The above data show that Aβ accumulation in a human CNS nerve cell line leads to the synthesis of proinflammatory cytokines and chemokines, elevated eicosanoid synthesis and the activation of inflammatory pathways. Prostaglandins tend to be neuroprotective and leukotrienes potentiate toxicity, whereas the activation of cannabinoid receptors prevents Aβ accumulation and toxicity. Both intracellular Aβ accumulation and nerve cell death are potentiated by 5-LOX metabolites and proinflammatory cytokines.

Age and chronic systemic inflammation are risk factors for many CNS diseases, including depression and Alzheimer’s,^26^ and the elevation of peripheral inflammation in old individuals frequently leads to cognitive decline.^27^ It follows that there is a complex interrelationship between nerve cell proteotoxicity, inflammation, aging and CNS disease.

Among CNS cytokines elevated in AD,^28^ all except IL6 are highly expressed in MC65 cells following Aβ induction. However, while there is an elevated inflammatory response within the AD brain, it has generally been assumed to result from the activation of microglia and astrocytes.^29,30^ Only in one report is a proinflammatory cytokine associated with nerve cells that contain Aβ.^31^ Here we outline the molecular basis of the proinflammatory program caused by the accumulation of intracellular Aβ.

Epidemiological data show that non-steroidal anti-inflammatory drugs delay the clinical features of AD,^32^ but clinical trials have failed.^33^ Perhaps this is because the drugs were COX inhibitors and, as shown here, the most toxic aspect of Aβ-induced inflammation is mediated by 5-LOX metabolites, whereas the COX metabolites PGE2 and PGD2 are neuroprotective. PGD2 is 100-fold more protective than PGE2. PGD2 is abundant in the CNS,^34^ and the observation that COX-2 expression is high in AD brain and in induced MC65 cells may be a reflection of the cells’ attempt at protection from Aβ toxicity.^35^

PGD2 activates the PD1 receptor, leading to an increase in cAMP. The activation of PD1 receptors is generally thought to be anti-inflammatory, and both PGD2 and the potent DP1 agonist, BW245C, reduce nerve cell loss in ischemic stroke models and excitotoxicity.^36^ PGE2 is also neuroprotective in several other neurodegeneration models.^37–39^ PGE2 functions through the EP receptors that are also coupled to cAMP production.

Of the multiple eicosanoids produced in the Aβ-initiated responses in MC65 cells, the leukotriene metabolites of 5-LOX are the only ones that potentiate toxicity. Curiously, only one 5-LOX inhibitor, Zileuton, has reached the clinic, and that is for the treatment of lung inflammation. Zileuton also reduces AD pathology in transgenic mice.^40^ Given the growing literature on the role of AA metabolism in AD, 5-LOX inhibitors may be therapeutically relevant. Excellent candidates are CNB-001 and fisetin, that are over 10-fold more potent than Zileuton and are very effective in AD transgenic mice.^14,41^

Caspase 1 is activated by Aβ in MC65 cells, and its activation can be initiated via pattern recognition receptors or RAGE.^42,43^ RAGE is highly expressed in neurons and is thought to be a receptor for Aβ. The inflammatory response to intracellular Aβ, as well as Caspase 1 activation and cell death, is reduced by the RAGE inhibitor FPS-ZMI and by its knockdown. Because there is neither a complete inhibition of cell death nor a complete reduction of intracellular amyloid by either the RAGE inhibitor or RAGE knockdown, it is unlikely to be the only pathway that contributes to MC65 cell death.

Endocannabinoids can be produced in response to stress,^44,45^ and in rodent AD models cannabinoids reduce Aβ accumulation and improve memory.^46,47^ THC also reduces inducible huntingtin overexpression in PC12 cells,^48^ and both THC and endocannabinoids reduce inflammation.^49,50^ Several synthetic, plant derived and endogenous cannabinoids are able to prevent the accumulation of intraneuronal Aβ, reduce the production of eicosanoids, and block nerve cell death. Therefore, it is reasonable to conclude that there is a therapeutic potential of cannabinoids for the treatment of AD.

**Figure 5:**
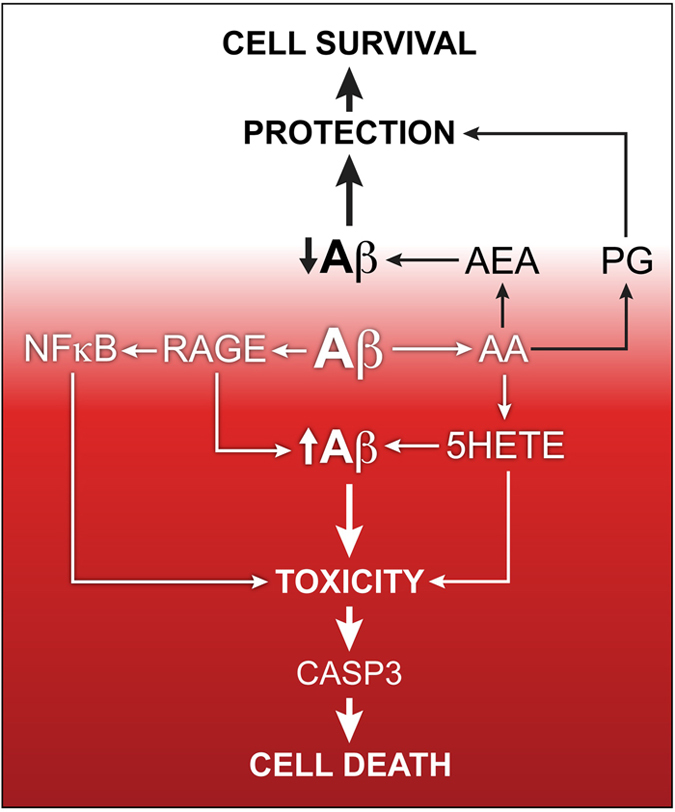
Summary of the multiple pathways that contribute to Aβ-induced nerve cell death. AA, arachidonic acid; CASP3, caspase 3; PG, prostaglandin; AEA, arachidonoyl ethanolamide.

In summary, the accumulation of intraneuronal Aβ and inflammation precede plaque formation and nerve cell death in AD. It is shown here that intracellular Aβ activates a broad spectrum of inflammatory signaling pathways. There is clearly a dynamic interplay between these pathways that may lead to either cell survival or death (Figure 5). Cell death can only be completely prevented by 5-LOX inhibitors, cannabinoids and caspase inhibitors. However, once the cell death process is underway, death can be reduced by some prostaglandins. Conversely, once initiated, cell death is potentiated by AA, LA, some leukotrienes, CB1/2 inhibitors and cytokines that enhance proinflammatory pathways. Despite these complexities, the data strongly suggest that early intervention via the reduction of intraneuronal Aβ proteotoxicity may reduce AD disease initiation or progression.

## Materials and methods

### Cell line and assays

The culture and induction of C99 in MC65 cells was performed as previously.^14^ Eicosanoids were assayed as described.^41^ Gene expression analysis was done by NanoString (Seattle, WA, USA) and cytokines assayed by Myriad RBM (Austin, TX, USA).

### SDS-PAGE and immunoblotting

Cultured cells were washed twice in cold phosphate-buffered saline, scraped into lysis buffer and proteins separated on 12% SDS-polyacrylamide gels.^14^ The following primary antibodies were used: 5-LOX (78 kDa), COX2 (74 kDa), cleaved caspase 1 (20,22 kDa), P-NFkB (70 kDa), NFkB (70 kDa) all from Cell Signaling (Danvers, MA, USA); Beta Amyloid (6E10; Wako, Richmond, VA, USA); actin (45 kDa); (Enzo Life Sciences, Farmingdale, NY, USA); 12-LOX (80 kDa; Cayman, Ann Arbor, MI, USA); 15-LOX (80 kDa; Cayman).

### Transfection of MC65 cells

MC65 cells were plated in 35-mm culture dishes and grown for 24 h. Medium was exchanged by 1 ml of fresh growth medium without serum and cells were transfected with 166 pmol of siRNA for hRAGE or a control siRNA (pooled, Santa Cruz, Dallas, TX, USA) using Lipofectamine RNAiMAX reagent (Invitrogen, Carlsbad, CA, USA). After 24 h cells were plated for experiments. The next day the cells were put into OptiMEM (Invitrogen) with or without tet for the various experimental paradigms.

## Figures and Tables

**Table 1:** Gene expression of cytokines and chemokines

*Name*	*Functions in inflammation*	*Ratio versus control*^a^		
		*D1*	*D2*	*D3*
CCL2	Chemokine, recruits monocytes	3.3	41	24
CXCL1	Cytokine, neutrophil chemoattractant	2.4	7.6	7.2
CXCL2	Cytokine, chemotactic to leukocytes	2.7	3.4	5.1
CXCL3	Cytokine, monocyte migration	1.8	4.2	6.0
IL-1-beta	Cytokine, induces COX-2, inflammation	1.8	5.1	4.4
Interferon-gamma	Cytokine, activates macrophages	1	1.2	7.2
TNF-alpha	Cytokine, stimulates acute phase reaction	3.3	5.1	3.2
iNOS	Inducible production of nitric oxide	1.1	2.4	8.6
TRL-6	Proinflammatory receptor	1	1.9	3.0
Fos	Transcription factor	1.1	3.8	4.1
Jun	Transcription factor	1.1	4.2	4.6
IL8	Chemokine, recruits neutrophils	5.0	35	58
IL8^b^	Secreted protein	ND	50^b^	139^b^

a Days 1, 2, and 3 following the induction of Aβ synthesis in MC65 human nerve cells. Ratio of induced to uninduced. Done by Nano-String. Twelve of 184 genes assayed.

b Secreted IL8 pg/ml/10^6^ cells.
