# Supplementary material for: Amyloid proteotoxicity initiates an inflammatory response blocked by cannabinoids
Source: NPJ Aging Mech Dis. 2016 Jun 23;2:16012–. doi: 10.1038/npjamd.2016.12 (PMC5514994; doi:10.1038/npjamd.2016.12)
Supplement: Supplementary Table S1 [file npjamd201612-s1.doc]

| **Supplementary table S1. Eicosanoid Pharmacology** | | | | |
| --- | --- | --- | --- | --- |
| **COX and LOX inhibitors** | **MOA** | **EC_50_ (μM)** | **Max**  **(Percent)** | **Toxicity** |
| Indomethacin | Nonspecific COX inhibitor | >20 | — | no effect |
| Ibuprofen | Nonspecific COX inhibitor | >20 | — | no effect |
| SC-560 | COX 1 Inhibitor | >10 | — | no effect |
| NS-398 | COX 2 Inhibitor | >10 | — | no effect |
| Ketorolac | Generic COX inhibitor | >10 | — | no effect |
| MK806 | 5-LOX inhibitor | 0.7 | 100 | protects |
| CNB-001 | 5-LOX inhibitor | 0.5 | 100 | protects |
| BWB70C | 5-LOX inhibitor | 0.2 | 100 | protects |
| **Lipases** |  |  |  |  |
| CAY10502 | cPLA2 Inhibitor | >10 | — | no effect |
| OBAA | Generic PLA2 Inhibitor | 1.1 | 56 | Inhibits |
| FkGk11 | iPLA2 Inhibitor | 0.5 | 72 | inhibits |
| MAFP | cPLA2 Inhibitor | >1 | — | no effect |
| Thioetheramide | sPLA2 Inhibitor | >2 | — | no effect |
| Chlorpromazine | Generic PLA2 inhibitor | 2.2 | 71 | inhibits |
| Pyrrophene | cPLA2 inhibitor | >10 | — | no effect |
| JZL184 | MAGL inhibitor | <20 | — | no effect |
| THL | Triglyceride lipase inhibitor | 1.5 | 40 | protects |
| RHC-80267 | Diacylglycerol inhibitor | >30 | — | no effect |
| CAY10499 | Generic lipase Inhibitor | 0.4 | 57 | protects |
| Orlistat | Generic lipase inhibitor | >5 | — | no effect |
| Atglistin | Triglyceride inhibitor | 0.5 | 38 | protects |
| FkGkll + THL | Combination |  | 100 | protects |
| **Eicosanoid Receptors** |  |  |  |  |
| SC 51089 | EP1 antagonist | 2.5 | — | potentiates |
| HA 23848 | EP4 antagonist | 0.2 | — | potentiates |
| GW 627368X | EP4 antagonist | 0.3 | — | potentiates |
| LY 255283 | BLT1 and BLT2 antagonist | >10 | — | no effect |
| BW 245C | PD1 agonist | 550 nM | 47 | protects |
| 15 PGJ (2) | PD2 agonist | — | — | no effect |
| BM567 | TP receptor antagonist | >10 | — | no effect |
| Bay U 3405 | DP2 antagonist | >10 | — | no effect |
| Bay U 9773 | LT inhibitor | >10 | — | no effect |
| **Cannabinoids** |  |  |  |  |
| AM 404 | AEA analogue | 0.4 | 80 | protects |
| Andandamide (AEA) | Endocannabinoid | 0.3 | 95 | protects |
| Cannabinoidiol | CB1 agonist | 0.1 | 56 | protects |
| THC | CB1 agonist | 0.05 | 100 | protects |
| AM 630 | CB2 antagonist | 0.01 | — | potentiates |
| AM 281 | CB1 antagonist | 0.9 | — | potentiates |
| Arvanil | CB1 agonist | 0.4 | 78 | protects |
| NADA | CB1 and CB2 agonist | 0.08 | 65 | protects |
| UBR 597 | Inhibits AEA degradation | 1.1 | 40 | protects |
| AM 251 | CB1 antagonist | 3.5 | — | potentiates |
| WIN-55, 212 | CB2 agonist | 1.5 | — | protects |
| JWH 133 | CB2 agonist | >10 | — | no effect |
| Q-3-Carboxamide | CB2 agonist | 0.09 | 93 | protects |
| AM 1241 | CB2 agonist | 0.30 | 100 | protects |
| MC65 cells were induced to make Aβ in the presence of three-fold serial dilutions between 10 nM and 20 μM of the indicated compounds. Cell viability was monitored daily, and the compounds either had no effect, potentiated Aβ toxicity at day 2 or 3, or inhibited Aβ toxicity at day 4. In the case of protection the maximum amount of protection is given, along with the EC_50_. In the case of inhibition the EC_50_ alone is indicated, There was no direct toxicity to uninduced cells at the concentrations indicated. At the indicted higher concentration (EC_50_>) the compounds were toxic to uninduced cells. | | | | |
