# Supplementary material for: Amyloid proteotoxicity initiates an inflammatory response blocked by cannabinoids
Source: NPJ Aging Mech Dis. 2016 Jun 23;2:16012–. doi: 10.1038/npjamd.2016.12 (PMC5514994; doi:10.1038/npjamd.2016.12)
Supplement: Supplementary Table S2 [file npjamd201612-s2.doc]

| **Supplementary table S2. Effects of Exogenous Eicosanoids** | | | | | |
| --- | --- | --- | --- | --- | --- |
| **Compound** | **No Effect** | **Protects** | **EC_50_** | **Potentiates** | **Potentiation**  **Low Dose** |
| Tetranar R12-HETE | + |  |  |  |  |
| Tetranar S12-HETE | + |  |  |  |  |
| 5 HETE |  |  |  | + | 450 nM |
| 12 HETE |  | + | 0.8 μM |  |  |
| 15 HETE | + |  |  |  |  |
| LTA4 |  |  |  | + | 300 nM |
| LTB4 |  |  |  | + | 75 nM |
| LTD4 | + |  |  |  |  |
| PGA2 | + |  |  |  |  |
| PGD2 |  | + | 70 nM |  |  |
| PGE2 |  | + | 8 μM |  |  |
| PGF2 | + |  |  |  |  |
| ± 9 HODE | + |  |  |  |  |
| 13 HODE | + |  |  |  |  |
| 9,10 diHOME | + |  |  |  |  |
| 12,13 diHOME | + |  |  |  |  |
| 8-isoPGA2 | + |  |  |  |  |
| 16-HDolte | + |  |  |  |  |
| 14,15 DiHETrE | + |  |  |  |  |
| 9(10) EpOME | + |  |  |  |  |
| 12,13 EpOME | + |  |  |  |  |
| 5 HpETE |  |  |  | + | 375 nM |
| 12 HpETE | + |  |  |  |  |
| 15 HpETE | + |  |  |  |  |
| The indicated eicosanoids were added to induced MC65 cells at time of induction at concentrations between 10 nM and 20 μM. Cell viability was determined at each day following Aβ expression. Compounds that potentiated toxicity were assayed on day 2 when there is no death of induced cells, and protection at day 4 when the induced cells were dead. None of the compounds were directly toxic to uninduced cells. | | | | | |
